# Genome-wide analysis of microRNA targeting impacted by SNPs in cucumber genome

**DOI:** 10.1186/s12864-017-3665-y

**Published:** 2017-04-04

**Authors:** Jian Ling, Zhongqin Luo, Feng Liu, Zhenchuan Mao, Yuhong Yang, Bingyan Xie

**Affiliations:** Institute of Vegetables and Flowers, Chinese Academy of Agricultural Sciences, 12 Zhongguancun South Street, Beijing, 100081 China

**Keywords:** Cucumber, MicroRNA, SNPs, Domestic selection

## Abstract

**Background:**

microRNAs (miRNAs) are endogenous small RNAs that play important regulatory functions in plant development. Genetic variations in miRNAs sequences or their target-binding sites (microRNA-target interaction sites) can alter miRNA targets in animal and human. Whether these single nucleotide polymorphisms (SNPs) in plant are functional have not yet been determined.

**Results:**

In this study, we constructed leaf, root, and stem-derived small libraries of cucumber (*Cucumis sativus*) line 9930 (cultivated China-group cucumber) and *C. sativus var. hardwickii* (wild India group cucumber). A total of 22 conserved miRNA families, nine less-conserved miRNA families, and 49 cucumber-specific miRNAs were identified in both line 9930 and *hardwickii*. We employed cucumber resequencing data to perform a genome-wide scan for SNPs in cucumber miRNA-target interaction sites, including miRNA mature sequences and miRNA-target binding sites. As a result, we identified a total of 19 SNPs in mature miRNA sequences and 113 SNPs in miRNA-target binding sites with the potential to affect miRNA-target interactions. Furthermore, we experimentally confirmed that these SNPs produced 14 9930-unique targets mRNAs and 15 *hardwickii*-unique targets mRNA for cucumber miRNAs. This is the first experimental validation of SNPs in miRNA-target interaction sites affecting miRNA-target binding in plants.

**Conclusions:**

Our results indicate that SNPs can alter miRNA function and produce unique miRNA targets in cultivated and wild cucumbers. Therefore, miRNA-related SNPs may have played important in events that led to the agronomic differences between domestic and wild cucumber.

**Electronic supplementary material:**

The online version of this article (doi:10.1186/s12864-017-3665-y) contains supplementary material, which is available to authorized users.

## Background

MicroRNAs (miRNAs), a class of 21–24 nt small RNAs, have important roles in post-transcriptional gene regulation in plants [1]. The precursors of miRNA genes have imperfect stem-loop structures and are further processed into miRNA-miRNA* by RNAIII-like nucleases. One strand of the duplex is associated with a member of the Argonaute (AGO) family. The resulting miRNA-AGO complex is the miRNA-induced silencing complex, which interacts with the complementary site of target mRNA transcript, thereby repressing the expression of the target transcripts at the post-transcriptional level [2].

The recently developed next-generation sequencing technology has provided a rapid and high-throughput tool to explore the large inventory of small RNA (sRNA) populations [3]. To date, a large number of miRNAs have been identified in diverse plant species [4–7]. The June 2013 release of miRBase 20 contains over 30,000 distinct mature miRNA sequences [8]. Many of the miRNAs are conserved in plants, while a large number of miRNAs show species specificity. The appearance in the database of large numbers of species-specific miRNAs (new miRNAs) suggests that miRNAs are born frequently but are also lost frequently, and the foldback sequences in genomes, inverted duplication events, and transposable elements, are thought to be important sources of many new miRNAs [9].

Genome-wide surveys of miRNA and miRNA binding site polymorphisms have been conducted widely in animals and human. These studies have shown that polymorphisms in miRNAs and their targets are less than in neutral regions. Further, the mutations at the miRNA and target sites exhibit a general signature of purifying selection [10]. Although, the polymorphisms of miRNA and miRNA binding site are low, several mutations can be detected in these loci, which may cause the change of miRNA targets. In animal and humans, many studies have reported that SNPs or indels in miRNA-related loci can change the mRNA targets of miRNA [11]. For example, 48 SNPs were identified in human miRNA seed regions and thousands of SNPs in the 3’ untranslated regions have the potential to either disturb or create miRNA-target interactions [12].

In plants, there were some reports on the miRNA and miRNA binding site polymorphisms in Arabidopsis and rice. These studied have proved that, like in animal and humans, both miRNAs and their target binding sites have low nucleotide variation and divergence compared to their flanking sequences, indicating strong purifying selection on these sites [13]. In plants, miRNA target recognition is highly dependent on interactions between complementary sequences in the miRNAs and their target sites in mRNAs (miRNA-target pair sites) [14]. Therefore, miRNA targeting and function can be affected by sequence polymorphisms in miRNA-target pair sites. In rice, 12 mutation sites were observed in the mature sequences of 11 miRNAs, and the SNPs in miR166 cause the production of new miRNA targets [13]. Although, these studies have shown how SNPs in miRNA-target pair sites affect miRNA targeting and function, there is still absent systematically studies on how SNP have impacts on miRNA-targets changes in plant.

In this study, we performed the deep sequencing of cucumber miRNA, and used re-sequence data to assess the variation of miRNA-related loci in cucumber. Our results showed that SNPs can alter miRNA function and cause unique miRNA targets for domestic and wild cucumbers. Therefore, miRNA-related SNPs could be an important factor that leads to the agronomic differences between domestic and wild cucumbers.

## Results

### Construction and sequencing of small RNA libraries

**Fig. 1:**
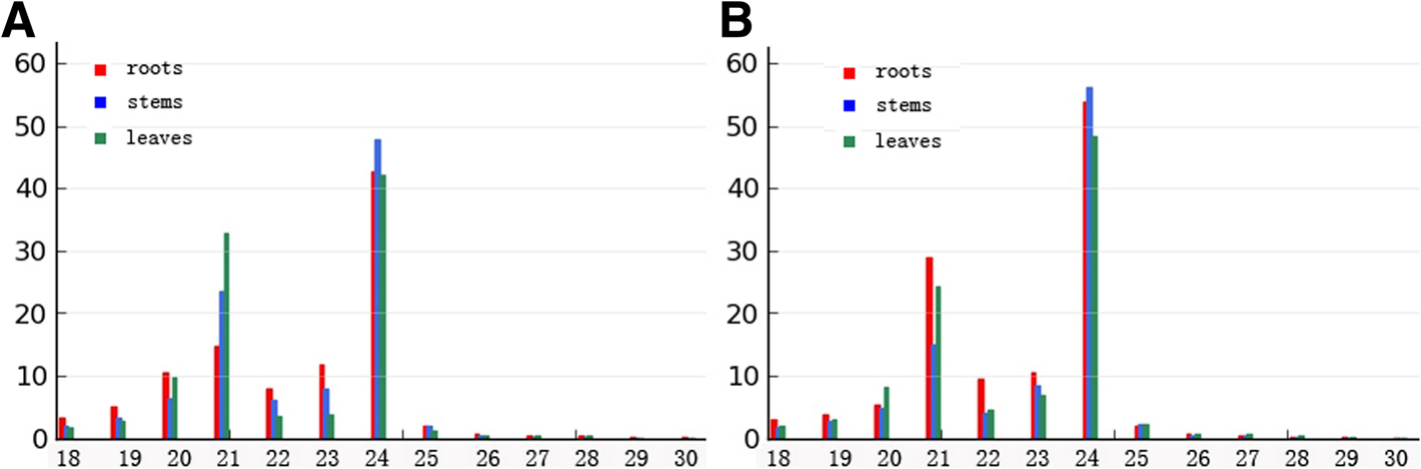
Size distribution of small RNAs in cucumber line 9930 and line *hardwickii.* **a**. Size distribution in 9930. **b**. Size distribution in hardwickii. The x-axis represent sRNA size (nt) distribution and the y-axis represent the frequencies (%) of size distribution

We constructed root, stem, and leaf-derived small RNA (sRNA) libraries for cucumber line 9930 and line *hardwickii*, respectively. For each library, on average more than 11 million clean reads and more than 3.5 million unique reads were obtained (Table 1). We mapped the unique reads to the corresponding line 9930 and *hardwickii* genomes. Averagely, more than 2.41 million of the non-redundant unique reads in each library had at least one perfect match to the cucumber genome, and were analyzed further. The size distribution of all the sRNA reads is summarized in Fig. 1. The cucumber sRNAs varied in length from 18 to 30 nt, and the majority were in the 20–24 nt range. The 24-nt long reads were the most abundant, followed by the 21-nt long reads, which is consistent with the findings in other plant species, such as *Arabidopsis*, rice and soybean.

**Table 1:** Statistics of short RNA sequences from the cucumber leaves, stems, and roots

		Leaves	Roots	Stems
TA	Line 9930	11406165	11849548	11867542
	*hardwickii*	11967608	10606465	11261249
US	Line 9930	3174202	3860476	3317204
	*hardwickii*	3625909	3837898	3519413
USM	Line 9930	2098673	2764366	2496933
	*hardwickii*	2106815	2969919	2027693

*TA*, total number of reads, *US* number of unique sequence, *USM* number of unique sequences matched to the genomes

### Identification of conserved and known miRNAs in cucumber

Conserved miRNA families have been found in many plant species and have been shown to have important functions in plant development and stress response [9]. We used the miRBase 20 database as a reference to identify conserved miRNAs in the six cucumber sRNA libraries. The reads with two or fewer mismatches to sequences in miRBase 20 were considered as candidate conserved miRNA. These candidate miRNAs were subjected to a rigorous secondary structure analysis using the Mireap software to predict their secondary structure [14]. Totally, we identified 22 conserved miRNAs families with canonical stem-loop structure (Additional file 1: Table S1). We refer to these sRNAs as conserved miRNAs in this study. We calculated the expression levels of conserved miRNA families based on RMP (reads per million reads) values (Table 2). Some of the conserved families were similarly high (e.g., miR156, miR166, and miR167) or low (e.g., miR393 and miR395) in all three tissues; however, some conserved miRNAs were more abundant in some tissues than in others. For example, miR162 expression in the root was more than three times its expression in stem and leaf. We also found some differences in miRNA expression patterns between 9930 and *hardwickii*. For example, in 9930, miR398 expression was higher in leaf than in stem and root, while, in *hardwickii*, the miR398 expression was low in all three tissues (Additional file 2: Figure S1).

**Table 2:** The abundance of conserved and known miRNAs family in cucumber tissues

	Lines 9930			*hardwickii*		
	Roots	Stems	Leaves	Roots	Stems	Leaves
Conserved miRNA						
miR156	41157.79	17310.32	34671.86	69197.32	26787.81	55323.58
miR159	245.42	267.45	440.81	170.31	177.06	267.73
miR160	627.02	405.39	274.5	43.93	49.12	55.23
miR162	135.23	32.1	15.43	130.26	35.35	46.20
miR164	918.65	1323.1	76.88	289.72	800.64	285.01
miR166	5334.08	12579.6	14272.63	15591.71	52495.52	62135.22
miR167	18016.33	4690.6	12650	3119.19	2667.24	16258.88
miR168	2306.63	3177.99	1745.72	5488.06	7501.65	5942.70
miR169	358.34	90.92	74.25	28.28	297.74	260.28
miR171	64.47	55.52	84.86	44.12	73.44	186.50
miR172	958.22	533.97	757.13	182.34	905.20	1639.42
miR319	24.04	79.79	0.96	26.90	70.15	1.68
miR390	149.46	139.54	40.5	7.16	71.46	87.82
miR393	2.67	5.14	23.32	3.48	3.72	10.27
miR394	1.15	10.19	16.21	3.20	22.53	30.66
miR395	11.91	0.25	0.08	1.12	0.06	0.08
miR396	563.33	188.83	704.53	88.43	179.79	633.29
miR397	0.57	53.17	75.22	7.25	0.03	0.09
miR398	0.07	27.89	67.94	2.04	0.34	0.07
miR399	18.12	599.61	211.9	45.82	100.41	80.46
miR408	33.50	60.58	123.61	48.92	0.06	0.23
miR827	31.98	539.2	601.69	42.61	3.11	10.36
miR1515	1.08	8.76	11.04	6.74	3.28	10.83
Known miRNA						
miR157	13362.55	35645.71	125371.8	10349.34	17252.43	64147.90
miR2111	31.40	66.98	11.22	23.62	76.54	22.22
miR2911	7415.80	828.56	951.76	681.09	79.65	9.76
miR2916	3.46	15.84	15.07	141.81	42.53	70.68
miR2950	235.09	42.71	1.86	4.80	20.24	4.42
miR4414	0.00	389.65	3.28	0.00	221.73	117.57
miR530	8.30	50.72	7.27	24.32	53.19	154.16
miR858	0.02	0.04	0.02	0.01	0.12	0.24

The RPM (reads per million reads) value was used to represent the expression levels of cucumber miRNA

In addition to the conserved miRNAs, there were other miRNAs that are not conserved, but that are found in only a few plant species. We refer to these less-conserved but known miRNA as known miRNAs in this study. A total of nine known miRNA families were identified in the six libraries (Table 2 and Additional file 1: Table S1). Most of the known miRNAs exhibited relatively low expression; however, a notable exception was miR157, which was expressed at an abundance of more than 10,000 reads per million (RPM) in all three tissues tested. Some of the known miRNAs displayed tissue-specific expression. For example, miR4414 expression was detected in the stem and leaf, but not in the root.

### Cucumber-specific miRNAs

After excluding the sRNA reads that were homologous to the conserved and known miRNAs, the remaining sRNAs with a maximum of 15 matches in the genome were subjected to rigorous secondary structure analysis of their precursors according to the criteria established by Meyers [15]. A total of 49 miRNA candidates contained stem-loop structure and met the screening criteria, and, for 18 of them, a miRNA star (miRNA*) sequence was identified in the same library. We considered the 18 miRNAs with miRNA* sequences as new cucumbers-specific miRNAs and the remaining 31 without miRNA* sequences as candidate cucumber-specific miRNA; collectively, we termed them cucumber-specific miRNAs (Additional file 3: Table S2).

To verify the existence and expression of the miRNAs identified in this study, we used real-time RT-PCR analysis to detect their relative expression pattern in root, stem and leaf between 9930 and *hardwickii* (Additional file 4: Table S3). The relative expression levels of 10 selected conserved, known miRNAs and 16 cucumber-specific miRNAs were analyzed. The results showed that the conserved and known miRNA showed fewer expression differences between 9930 and *hardwickii* than the cucumber-specific miRNAs (Additional file 4: Table S3).

### SNPs and nucleotide divergence in cucumber miRNAs

**Fig. 2:**
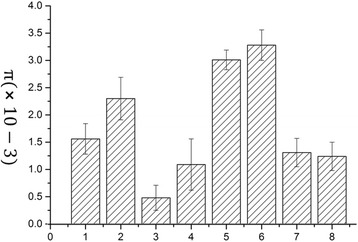
The nucleotide diversity of miRNA-related loci in cucumber. The average pairwise nucleotide diversity (π, y-axis) is used to analyzed the nucleotide diversity of cucumber miRNA-related loci. X-axis is represented different miRNA-related loci (1: conserved and known miRNA loci; 2: cucumber-specific miRNA; 3: mature miRNA sequences of conserved and known miRNA; 4: mature miRNA sequences of cucumber-specific miRNA; 5: 2 kb up and down stream of conserved and known miRNA; 6: 2 kb up and down stream of cucumber-specific miRNA; 7: miRNA-mRNA interaction sites of conserved and known miRNA; 8: miRNA-mRNA interaction sites of cucumber-specific miRNA)

In this study, we used the cucumber resequencing data to assess genome-wide patterns of nucleotide diversity for the cucumber miRNAs. A whole-genome SNP analysis, with the parameter π (average pairwise nucleotide diversity), was used to identify nucleotide diversity in the loci of the miRNA genes. The average π values of the conserved miRNA and cucumber-specific miRNA genes loci were 1.56 × 10-3 and 2.3 × 10-3 respectively (Fig. 2), which is significantly lower (*p* < 0.001) than the π value of the cucumber genome (3.17 × 10-3), indicating that the miRNA loci are more conserved than the cucumber genome as a whole. We also analyzed the nucleotide diversity of miRNA-related loci, including miRNA mature sequences loci, miRNA gene loci (miRNA precursor sequences), miRNA flanking regions (2 kb up-stream and down-stream of miRNA loci) and miRNA-target binding sites. As expected, the highest nucleotide diversity was observed for the miRNA flanking regions, followed by the miRNA gene loci. Except for the miRNA flanking regions, other miRNA-related loci showed significantly low sequence diversity compare to cucumber genome (*p* < 0.001). Notable, the nucleotide diversity of miRNA-target interaction sites, including miRNA mature sequences and miRNA-target binding sites was much lower than that of miRNA gene loci, indicating that these loci were much conserved in the evolution.

### miRNA target alteration by SNPs in miRNA mature sequences

In plant, microRNA target recognition is highly dependent on sequence complementarity between miRNA mature sequences and their target. Therefore, miRNA targeting and function can be affected by polymorphisms in those sequences. We first used cucumber resequencing data to identify the SNPs in cucumber miRNA mature sequences. Totally, for conserved and known miRNA, only four miRNA mature sequences, miR164a, miR166b, miR399b and miR396d were actually found to be polymorphic (Table 3). Compared with conserved and known miRNAs, we detected more SNPs in cucumber-specific miRNA mature sequences, with 15 SNPs for 13 cucumber-specific miRNAs (Table 3). To analyze the effects of SNP on miRNAs function, we constructed snp-miRNAs corresponding to their cognate miRNAs according to resequencing data. Two miRNA target finding software, mentioned in our material and methods, were used to predict targets for the both snp-miRNAs and their cognate miRNAs. Our results showed that snp-miRNAs could interact with a total of 34 new targets compared with the cognate miRNAs (Additional file 5: Table S4), with snp-miRNAs 396d having the maximum number (up to nine) new targets. Finding of lots of new targets for snp-miRNAs suggest that SNPs in mature sequences could cause miRNA to obtain new functions.

**Table 3:** The SNPs within miRNA mature sequences and their frequencies

miRNA	Snp position*	Base frequency (%)*
miR164a	14	C(40%);G(56%);-(4%)
miR166	4	G(83%);A(9%);R(8%)
miR395	20	C(91%);S(4%);-(4%);G(1%)
miR396d	16	C(94%);G(4%);-(1%);-(1%)
csa-mir4	6	G(96%);-(3%);A(1%)
csa-mir11	21	T(71%);C(24%);Y(3%);-(2%)
csa-mir22	3	G(98%);A(2%)
csa-mir24	6	A(92%);R(7%);-(1%)
csa-mir27	18	C(90%);Y(9%);-(1%)
csa-mir27	21	G(91%) K(9%)
csa-mir33	15	T(83%);K(6%);G(4%);-(7%)
csa-mir38	2	A(64%);G(21%);R(10%);-(5%)
csa-mir39	12	T(96%);-(2%);C(2%)
csa-mir40	12	C(60%);T(32%);R(4%);-(4%)
csa-mir40	15	C(77%);T(18%);-(4%);-(1%)
csa-mir42	14	T(91%);C(6%);R(3%)
csa-mir43	6	C(98%);M(1%);A(1%)
csa-mir44	8	T(92%);C(8%)
csa-mir48	10	G(76%);A(20%);R(4%)

* the snp position from 5’ end of corresponding miRNA

* alphabets that are not belong to any of A,C,G and T represent degenerate bases

* - represent indels

**Fig. 3:**
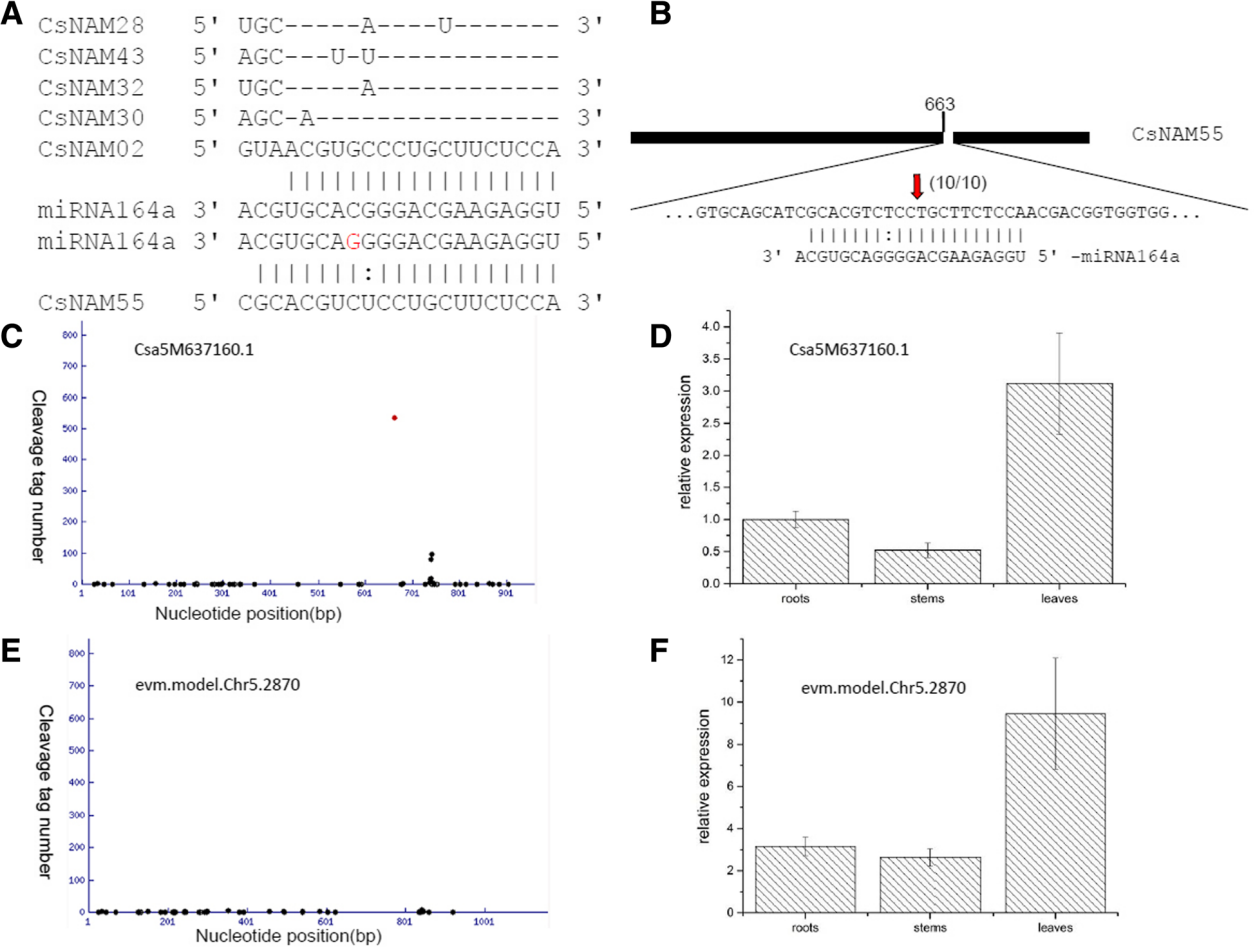
The targets analysis for miR164 and snp-miR164 in domestic cucumber. **a**. predicted targets for miR164 and snp-miR164. The short vertical bars represent match base pair and two vertical dots representing a G-U pair. **b**. a schematic figure represents the interaction between snp-miRNA164a and it’s predicted target *CsNAM55*. *Thick black line* represents mRNA of CsNAM55. Number on the *black line* represents cleavage site within mRNA, which also was represented by the *red arrow*. The snp-miRNA164 and the target mRNA are showed in the expanded regions. The number in the parentheses represents the number of sequenced 5’ RACE clones corresponding to each site. **c**. Target plots of snp-miRNA164 target CsNAM55 confirmed by degradome sequencing. The *black dots* represent degrade tags from degradome sequencing. The *red dot* represents the most abundant cleavage tag derived from miRNA cleavage site. **d**. The expression of CsNAM55 revealed by real-time PCR. β-actin was used as an internal control to normalize the data. For compare analysis, we set expression of CsNAM55 in line 9930 root as control. **e**. Target plots of snp-miRNA164 target CsNAM55 confirmed by degradome sequencing. The *black dots* represent degrade tags from degradome sequencing. The *red dot* represents the most abundant cleavage tag derived from miRNA cleavage site. **f**. The expression of evm.TU.Chr5.2870, which is an orthologous gene of CsNAM55 in line hardwickii, compared with expression of CsNAM55 in line 9930 roots

It was noted that miR164a SNP showed a significant difference between China-group and India-group cucumber. In India-group cucumber, all accessions have the same base (C) in the 14th base from 5’ end of miRNA164a, whereas about half of China-group cucumber (14/37) has base G in the locus (here we referred to as snp-miR164). The Snp-miR164 sequence was detected in 9930 sRNA libraries. We used degradome data to confirm the targets for miRNA164 and snp-miR164. As showed in Fig. 3a, we identified five targets of miRNA164, and all five of these mRNAs encoded members of the NAM (no apical meristem) protein family. It should be noted that snp-miR164 had a unique target named *CsNAM55* (*Csa5M637160.1*) that also belongs to the NAM protein family, indicating that the SNP cause miRNA164a to obtain a new target. The cleavage site of snp-miR164 was at 663 bp from 5’-end of *CsNAM55*, which corresponding to our degradome data (Fig. 3b, c). However, no statistic significantly cleavage site for *evm.model.Chr5.2870*, the orthologous genes of *CsNAM55* in line *hardwickii*, can be detected (Fig. 3e), indicating that miR164 is unlikely to cleave *evm.model.Chr5.2870*. The expression levels of *CsNAM55* detected by real-time PCR were lower than that of *evm.model.Chr5.2870* in all tissues tested (Fig. 3d, f), suggesting that *CsNAM55* expression was repressed by snp-miR164. NAM proteins are plant-specific transcription factors involved in development processes, such as formation of the shoot apical meristem and lateral shoots. The ability of Snp-miR164 to target a unique NAM gene suggests that a new regulatory role for miRNA164 in domestic cucumber development.

### miRNA target alteration by SNPs in miRNA-target binding sites

We used 9930 mRNA genes to predict targets for cucumber miRNAs, and found a total of 392 and 231 mRNAs that could be the candidate targets for the conserved and cucumber-specific miRNA, respectively (Additional file 6: Table S5). We identified the SNPs at miRNA-mRNA binding sites by using the resequencing data. We detected a total of 56 and 57 SNPs in the miRNA-target binding sites of conserved and cucumber-specific miRNA, respectively (Additional file 7: Table S6), suggesting these SNPs could affect miRNA-target binding. To analyze the influence of SNPs in miRNA-target binding sites on miRNA/target interactions, we focused on the SNPs differences of miRNA-target loci between 9930 and *hardwickii*. We detected 35 miRNA-target binding sites SNPs between 9930 and *hardwickii* for conserved and known miRNA, and 31 for cucumber-specific miRNAs (Additional file 8: Table S7). To confirm whether those SNPs be functional SNPs, according to SNPs at miRNA-target binding sites and 9930 mRNA sequences, we reconstructed snp-mRNA sequences corresponding to their cognate mRNA in 9930, and we also used two kinds of softwares to find miRNA targets in snp-mRNA sequences. In addition, to confirm the reconstructed snp-mRNA sequences being derived from *hardwickii* mRNA, we used the snp-mRNA sequences blast to *hardiwkki* annotated mRNA. Those snp-mRNA sequences were considered as potential *hariwikki* mRNAs, if they satisfied the following strict criteria: (1) the similarity between the snp-mRNA sequences and *hardwickii* mRNAs is higher than 90% (2) the corresponding *hardwickii* mRNA must contain predicted miRNA-mRNA binds sites of snp-mRNA. We referred to these snp-mRNA sequences as *hariwikki* mRNAs. We compared the target differences between 9930 mRNA and *hardiwkikki* mRNA, for conserved and known miRNA, we found that the miRNA-mRNA binds sites SNPs between 9930 and *hardwickii* disrupted 20 miRNA-mRNA complementary binding sites in 9930, and created 24 new potential target binding sites in *hardwickii*, which potentially creating 20 9930-unique and 24 *hardwickii*-unique miRNA targets (Additional file 8: Table S7). For cucumber-specific miRNA, we detected 24 9930-unique and 22 *hardwickii*-unique target miRNAs (Additional file 8: Table S7).

### Experimentally confirm miRNA target alteration by SNPs in miRNA-mRNA binding sites

**Fig. 4:**
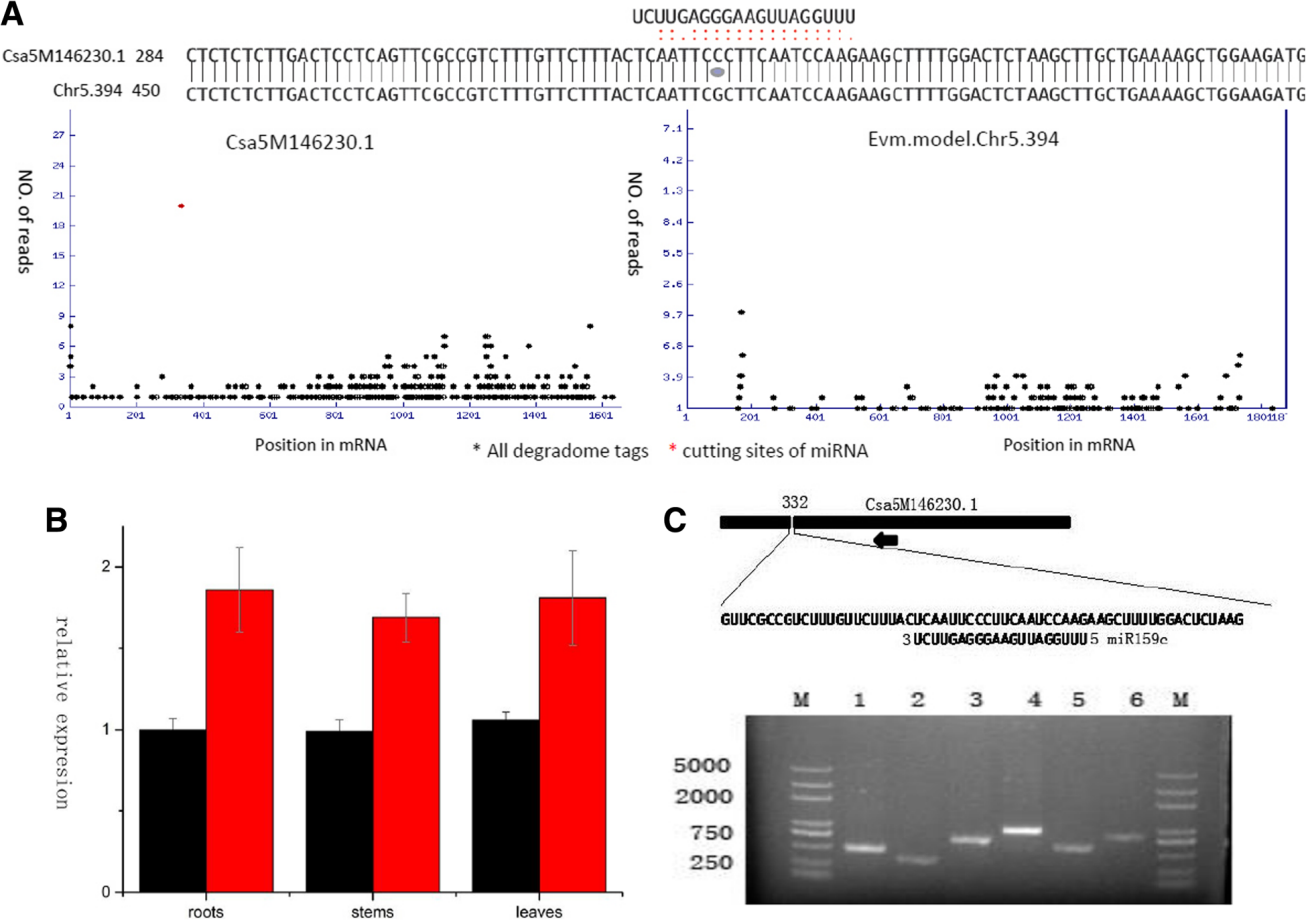
Experimentally confirm SNPs-causing 9930-unique target *Csa5M146230.1.* **a**. 9930-unique target *Csa5M146230.1* for miRNA159c confirmed by degradome sequencing. The base pair of miRNA159c with *Csa5M124230.1* and the base pair of *Csa5M124230.1* with its orthologous hardwickii gene *evm.model.Chr5.394* were showed on the top. The *red vertical* two points represent match base pair and one *red vertical dot* representing a G-U pair between miR159c and Csa5M124230.1. The *black vertical bars* represent the same base between Csa5M124230.1 and evm.model.Chr5.394. The black circle represents a SNP. The degradome sequencing data is plotted on the bottom. The *left* plot represents degradome tags distributing on mRNA of Csa5M124230.1(*black dots*), and the *red dot* represents the most abundant cleavage tag derived from mRNA cleavage site according to related statistical analysis of Cleaveland 2.0 software. The *right* plot represents degradome tags distributing on mRNA of evm.model.Chr5.394, and no statistical significant cleavage site can be detected. **b**. Real-time PCR analysis for Csa5M124230.1 (black columns) and evm.model.Chr5.394 (*red columns*) in roots, stems and leaves of 9930 and hardwickii, respectively. SE bar are shown. The y-axis representing relative expression compared to 9930 roots. **c**. Diagrammatic representation of *Csa5M124230.1* cleavage sites are showed on the top. *Thick black line* represents mRNA of *Csa5M124230.1*. The putative miRNA interaction site is shown as a *white box*, with the nucleotide position within mRNA indicated. The miRNA sequence and partial sequence of Csa5M124230.1 are shown in the expanded regions. Horizontal arrowhead indicates gene-specific primer sites used for 5’ RACE. On the bottom, ethidium bromide-stained agarose gel showing the 5’RACE for six selected 9930-unique targets (1: *Csa5M124230.1*;2:*Csa6M040600.1*;3:*Csa4M016490.1*;4:*Csa1M029600.1*; 5: *Csa6M423430.1*; 6: *Csa4M290150.1*) listed in the additional file 9, and the number one corresponding to 5’ RACE reactions specific for Csa5M124230.1. Lane M, 2 k-plus ladders

We further used experimental methods, including degradome analysis, 5’RACE and realtime PCR, to confirm these unique miRNA targets. As showed in Fig. 4, the degradome data of line 9930 indicated that miRNA159c has a cleavage site at 333 bp within mRNA of *Csa5M146230.1*(Fig. 4a), and this target can be classed into category 0 as a result of the most abundant cleavage tags can be detected at the cleavage site, according to category criteria established by Cleaveland 2.0. However, by analyzing the degradome data of line *hardwickii*, we failed to found a statistical significant cleavage site for *hardwickii* gene *evm.model.Chr5.394*, which is a high similar (99% in nucleotide level) orthologous gene for *Csa5M146230.1*. A nucleotide difference between *Csa5M146230.1* and *evm.model.Chr5.394* can be detected at 331th bp site of *Csa5M146230.1* (Fig. 4a), and our degradome results suggested that this SNP is likely to affect miRNA-mRNA binding by disrupting the base pair between miRNA159c and its target. We further employed 5’RACE to verify the cleavage site within *Csa5M146230.1* by using a gene-specific primer designed at 792 bp site of *Csa5M146230.1*, and we obtained a 484 bp band (Fig. 4c), which were consistent with our degradome sequencing data. By comparing expression levels of *Csa5M146230.1* and *evm.model.Chr5.394* in different tissues, we found that the expression levels of *Csa5M146230.1* were lower than those of *evm.model.Chr5.394* (Fig. 4c), indicating that miRNA159c inhibit the expression of *Csa5M146230.1* but not for *evm.model.Chr5.394*. Together, our experiments confirmed miRNA159c can cleavage *Csa5M146230.1* but not for *evm.model.Chr5.394*.

Totally, we experimentally verified six 9930-unique targets and nine *hardwickii*-unique targets for conserved cucumber miRNA (Table 4), including some members of transcriptional factor such as GRAS, F-box and so on, suggesting that these 9930-unique or *hardwickii-*unique targets could play roles in various differences between domestic and wild cucumbers. Our real-time PCR analysis showed that most of unique targets expressions were inhibited by miRNA compared to their corresponding orthologus genes that were no more than the targets for the miRNA (Additional file 9: Figure S2. Some exceptional cases were also observed. For example, *hardwikki*-unique *evm.model.chr6.523* was the target of miRNA408. The expressions of *evm.model.chr6.523* were higher than those of its orthologous gene in 9930(Additional file 9: Figure S2. In addition, we also experimentally verified eight 9930-unique targets and six *hardwickii-*unique targets for cucumber-specific miRNAs (Additional file 9: Figure S2).

**Table 4:** SNPs-causing 9930-unique targets and *hardwickii-*unique targets confirmed by experiments

miRNAs	targets	Cleavage site	Function annotation
9930-unique targets			
miR159c	*Csa5M146230.1*	332	TMPIT
miR171b	*Csa6M040600.1*	399	GRAS
miR2111	*Csa4M016490.1*	918	Transferase
miR398a	*Csa2M295970.1*	1688	PPR
miR399a	*Csa4M001720.1*	1378	Glyco_hydro_1
miR858	*Csa6M517090.1*	1160	Pkinase_Tyr;LRR_1
csa-mir13	*Csa6M423430.1*	2860	RRM_2
csa-mir17	*Csa4M303180.1*	542	UDPGT
csa-mir24	*Csa6M361370.1*	1561	dsRNA_bind;PAZ;helicase_dead;Ribonuclease_3
csa-mir27	*Csa7M396360.1*	1627	ALG3
csa-mir29	*Csa1M043010.1*	427	Hydrolase_4
csa-mir45	*Csa4M290150.1*	438	Pkinase_Tyr;B_lectin
csa-mir46	*Csa1M029600.1*	252	EMP70
csa-mir7	*Csa3M852500.1*	1096	
*Hardwickii*-unique targets			
mir156e	*evm.model.Chr3.3319*	461	UDPGT
mir169b	*evm.model.Chr3.3978*	552	F-box
mir169b	*evm.model.Chr5.1830*	819	DUF26
mir171a	*evm.model.Chr2.1408*	927	SET
mir396a	*evm.model.Chr6.793*	725	PPR
mir396b	*evm.model.Chr6.762*	254	Pro_isomerase
mir398b	*evm.model.Chr3.334*	965	HMA;Sod_Cu
mir398b	*evm.model.Chr7.266*	4582	PAS_2;Phytochrome;GAF;HATPase_c;PAS
mir408	*evm.model.Chr6.3144*	924	Ras
csa-mir11	*evm.model.Chr2.2052*	1853	DUF3490;Kinesin
csa-mir12	*evm.model.Chr1.775*	1845	Aldedh
csa-mir18	*evm.model.Chr2.326*	1851	Pkinase
csa-mir18	*evm.model.Chr7.634*	1355	HLH
csa-mir20	*evm.model.Chr3.488*	489	MoeZ_MoeB;ThiF;Rhodanese
csa-mir25	*evm.model.chr6.523.2*	383	

## Discussion

### Cucumber miRNAs and their genetic diversity

In this study, we identified a total of 31 conserved and less-conserved miRNAs (22 conserved and nine less-conserved), which the nomenclature of cucumber miRNA was obtained according to the criterion of miRNA research [16]. Compared with the results of previous study in cucumber miRNA [6, 17], there were some differences in our results. For example, we identified five new conserved or less-conserved miRNAs (miR2916, miR157, miR2911, miR4414 and miR395) in cucumber that were not detected in the studies in previous study. The different results may be partly explained by differences between the tissue-derived sRNA libraries that were used. The libraries used in the Martinez’s study were derived from phloem and leaves of cucumber, and the libraries used in the Mao et al. study were derived from leaves and roots. In our study, the libraries were derived from the stem, leaf and root. It is likely that the different miRNAs will have different expression levels in the various tissues and, therefore, some miRNAs may be detected in one tissue, but not in other tissues. We also identified 49 new miRNAs in this study and each of these 49 new miRNAs was predicted to have a canonical stem-loop structure. In the previous two studies, a total of nine new cucumber miRNA were identified [6, 17]. Mao et al. identified two new cucumber miRNAs, csa-miRn1-5p, which corresponds to our csa-miR8, and miRn2-5p, which corresponds to our csa-miR10. Martinez et al. identified six new cucumber miRNAs, including csa-mir3, which corresponds to our csa-miR4, and csa-mir5. Due to many New miRNA derived from repeat regions, therefore, the prediction result for new miRNAs also variable [1].

Qi et al. reported that genome-wide nucleotide diversity of domestic cucumbers was significantly lower in domestic cucumbers compared with wild cucumbers [18], and our analysis of between the domestic and wild cucumber libraries confirmed this earlier finding. Our results showed that domestic selection had reduced the genetic diversity of miRNA loci compared with the genetic diversity of miRNA loci in wild cucumber. Although, the nucleotide diversity of domestic cucumbers is significantly lower than that of wild cucumbers, the domestic cucumbers have group-specific SNPs. For example, our results indicated that the 9930-specific SNPs occurred at the 14th base in the mature miR164a sequence. Our study also showed that the SNP could produce new mRNA targets for miR164a. These results indicated that some variations have been fixed in cucumber miRNA loci by domestic selection (Additional file 10: Figure S3) However, how miR164a regulates the expression of its target genes is still unknown.

### SNPs play an important role in the expressions of miRNA and their targets

miRNAs regulate plant growth and development, and respond to various stresses through negative, post-transcriptional regulation of the expression of their target genes [19–21]. At the same time, miRNAs expression is also regulated in tissue-specific, developmental-specific and stresses-induced manner. For example, our results indicated that even several conserved miRNA showed a differential expression in different tissues. We also found some differences in the miRNA expression patterns between 9930 and *hardwickii*. For example, the expression of miR398 in 9930 showed an obvious difference compared with in *hardwickii*, suggesting that the abundance of several miRNAs of closely related cucurbit species was also distinctly different, which is similar with previous report [22].

SNPs in miRNA seed regions and translated regions have potential to change the miRNA expression. Several SNPs in human miRNA seed regions and untranslated regions are supposed to have the ability to change miRNA expression [12]. SNPs in the mature sequences of 11 rice miRNA have possible impacts on the miRNA expression [13]. MiRNAs, like protein-coding genes, have their own promoter region. The SNPs in promoter region of protein-coding can change the gene expression. Therefore, the SNPs in promoter region of miRNA could also change miRNAs expression. Many evidences suggest that SNPs in human miRNA genes promoter can alter the expression of miRNA [12]. We next will investigate the association between the SNPs and miRNA expression in cucumber.

The genetic diversity, including SNP and indel, within miRNA-targets interaction sites can affect the recognition and binding between miRNAs and their targets. In this study, we found a total of 19 SNPs within mature sequences and 113 SNPs within binding sites, suggesting the SNPs could impact miRNA-targets binding. We experimentally confirmed that the SNPs disturbed or created miRNA-binding that cause to 9930-unique or *hardwickii*-unique targets. On the other hands, we found that most of SNPs within miRNA-targets interaction sites had no influence on the binding despite of these SNPs have decreased or increased the complementarity between miRNA and targets. So a question rose. What degrees of complementarity within miRNA-targets interaction sites decided the reorganization between miRNAs and their targets, or decided the transcriptional repression and transcriptional cleavage. In animal, the complementarity between 6 bp seeds region of miRNAs and their binding sites are required for the recognition. The SNPs or indels outside of the seeds regions have little impact on bindings, while within the seeds regions the SNPs and indels will exclusively alter miRNA targets. Whether is there seeds regions within plant miRNA mature sequences? More studies on how miRNAs interact with their targets should be carried on.

### SNPs associated with important agronomic trait in cucumber

Cultivated and wild cucumbers show significant differences in various agronomic traits. Most of these agronomic traits are regulated by miRNAs such as miR156 and miR164, and many transcriptional factors are the targets of miRNAs. MiRNAs have an important regulatory function in plant development and, therefore, may have important roles in regulation pathways that contribute to the significant differences in various agronomic traits between cultivated and wild cucumbers. In our study, based on a genome-wide degradome analysis, we identified many 9930-unique and *hardwickii*-unique target mRNAs of cucumber. Some of the unique targets were the members of transcriptional factor. For example, NAM proteins are plant-specific transcription factors involved in development processes, such as formation of the shoot apical meristem and lateral shoots. In our study, one of NAM protein (CsNAM55) is the target of snp-miR166, and this result was found only in domestic cucumbers. Interestingly, NAM protein family is also underwent domestic selection in domestic cucumber. Our results suggest that the changes in miRNA targets would have resulted from domestic selection.

To verify whether the miRNA-related SNPs have agronomic importance, we perform GWAS analysis for this SNPs. Cucumber fruit length (fl) showed an obvious difference between 9930 and *hardwickii*, which is a typical agronomic trait of domestic selection. We found a SNP (snp40941) occurred in the miRNA-target interaction sites of miRNA408 and its target Csa3M214580.1 shows a significant association with fl (*p* < 0.01). It was noted that the snp40941 located in a selective sweep region of Chromosome 3 which is associated with fl. Therefore, our study suggests that the miRNA-related SNPs could have important impact to crop agronomic traits.

## Conclusions

We identified 22 conserved miRNA families, nine less-conserved miRNA families, and 49 cucumber-specific miRNAs in cucumber genome. Using cucumber resequencing data, 19 SNPs in miRNA mature sequences and 113 SNPs in miRNA-target binding sites were identified. We found that SNPs can alter miRNA function and cause unique miRNA targets for domestic and wild cucumbers, which leads to the agronomic differences between domestic and wild cucumber.

## Methods

### Plant materials

The ‘Chinese long’ inbred line 9930, a typical cucumber of northern China, was used to represent China-group cucumbers. *Cucumis sativus var. hardwickii* (PI: 183967) was used to represent wild India-group cucumbers. In addition, five selected China-group cucumbers and five selected India-group cucumbers are shown in Table 5. Cucumber seeds were germinated in pots containing vermiculite, and 3-week old seedlings were used to DNA isolation. The roots, stems, leaves, cotyledons of seedlings were collected separately for RNA isolation.

**Table 5:** The China-group and Inida-group cucumbers used in our study

Name	PI	Origin
Bei Jing Xiao Ci	V05A0674	Beijing, China
He_Cha	V05A0920	Hebei, China
Qian Qi Li Huang Gua	V05A1115	Hebei, China
Ye San Bai	V05A0985	Hebei, China
Fu Song Ye San	V05A0334	Jilin, China
LJ 90430	Ames 28156	India
VIR 3147	PI 504564	India
Small Green	PI 504570	India
10382	PI 175120	india
9779	PI 173892	india

### Small RNA library construction and sequencing

The roots, stems and leaves of cucumber 9930 and *hardwickii* were collected at the two-leaf stage, respectively. The total RNA was isolated by TransZol up plus RNA Kit (Transgen) according to the manufacturer’s instructions. About 100 μg of small RNA were used for sequencing by Beijing Genomics Institute (BGI) (Shenzhen, Guangdong, China) following the manufacturer’s protocols. In brief, sRNAs with 10-40 nt were isolated by 15% den aturing polyacrylamide gel electrophoresis. After ligating with 5’ and 3’ adaptors, the sRNA with 5’ and 3’adaptors were reversely transcribed to cDNA by using Superscript II reverse transcriptase (Invitrogen). The resulting cDNA were sequenced by the Illumina 1 G Genome Analyzer.

### Identification of conserved, known and cucumber-special miRNAs

After filter the adapter sequences, low quality sequences, the remaining small RNA with 16-30 nt were further subjected to mRNA, RFam, Repbase filter. Finally, the remaining unique sequences were used to do a blastn search against the miRbase 20 (http://www.mirbase.org/) to identify the conserved and known miRNAs in cucumber. A maximum of two mismatches were allowed between identified cucumber miRNAs and currently known plant miRNAs. To identify potential cucumber-special miRNAs, the remaining small RNA sequences firstly were mapped to cucumber genome by Bowtie2 [23]. Then the mireap pipeline (https://sourceforge.net/projects/mireap/) was employed to identify cucumber-special miRNAs. The small RNA sequences, which met previously described criteria [15], were then considered to be a potential cucumber-special miRNA.

### Expression of miRNA by Real-time PCR analysis

In this study, the expression of miRNAs was verified by qRT-PCR. A miRcute miRNA qPCR Detection kit (SYBR Green) (FP401; Tiangen) was used for qRT-PCR, and small U6 snRNA served as an internal reference. Primers for qRT-PCR are listed in Appendix S1. We performed real-time PCR using BIO-RAD CFX96 (Bio-Rad, USA). Amplification first begin with an initial 94 °C for 120 s, followed by 44 cycles of 94 °C for 30 s, 60 °C for 34 s and 30 s at 72 °C. The final melt step of 95 °C for 55 s, 56 °C for 40 s and 95 °C for 30 s. Cucumber U6 snRNA (*Csa3M314760.1*) was used as an internal reference. Three biological replicates were carried out. The ΔΔCT method was used to calculate the relative expression between 9930 and *hardwickii* [24].

### Snp and diversity analysis

The sequenced 9930 genome was used as a reference genome [25]. The resequencing data of various cucumbers (http://cmb.bnu.edu.cn/Cucumis_sativus_v20/) were obtained from cucumber genome database. Summary statistics parameter (π) for nucleotide diversity was calculated with a perl PopGen module.

### PCR and DNA Sequencing

We used the Primer3 (http://primer3.sourceforge.net/) to design the primers pairs to amplify precursor sequences of miRNA genes according to the genomic sequence of cucumber 9930 and *hardwikki.* Details of the primers were provided in Additional file (Additional file 11: Table S8). Amplification consisted of 30 cycles each as 30 s at 94 °C, 30 s at 55 °C, and 30 s at 72 °C. The denatured step in the first cycle was 3 min and the extension step in the last cycle was 10 min. The PCR products were directly sequenced using the forward or reverse primer, or were cloned into pEASY-T1 Cloning Kit (Transgen) and sequenced using the forward or reverse primer.

### miRNA-target prediction, degradome library construction and target identification

We predicted the target genes of miRNA by psRNATarget (http://plantgrn.noble.org/psRNATarget/) [26] and a perl script named axtell_targetfinder.pl that developed by Mike Axtell [27]. The degradome analysis and the classification of target categories were performed using the CLEAVELAND pipeline 2 [27].

### Validation of target genes by 5’ RACE

We carried out 5’ RACE using 5’-Full RACE Kit (Takara) according to the manufacturer’s instructions. Details of the primers were provided in Additional file 12: Table S9. The products were cloned into pEASY-T1 Cloning Kit (Transgen) and sequenced using the forward or reverse primer.

### Real-time PCR analysis of target genes

The real-time PCR analysis was performed according to Ling et al., [24]. Briefly, we performed real-time PCR using BIO-RAD CFX96 (Bio-Rad, USA). Amplification consisted of 40 cycles each as 30 s at 94 °C, 30 s at 55 or 60 °C, and 30 s at 72 °C. The denatured step in the first cycle was 3 min and the extension step in the last cycle was 10 min. Three biological replicates were carried out and triplicate quantitative assays for each replicate were performed. The cucumberβ-actin gene was used as an internal control. The primers used were listed in Additional file 13: Table S10.

## Additional files

Additional file 1: Table S1. Conserved and known miRNA of cucumber. (XLSX 15 kb)

Additional file 2: Figure S1. The heat map of miRNAs expression. (PDF 9 kb)

Additional file 3: Table S2. Cucumber-specific miRNA. (XLSX 22 kb)

Additional file 4: Table S3. The primers and relative expression of cucumber miRNA. (XLSX 10 kb)

Additional file 5: Table S4. The SNPs within miRNA mature sequences and their predicted targets. (XLS 23 kb)

Additional file 6: Table S5. The predicted targets for conserved and cucumber-specific miRNA. (XLSX 55 kb)

Additional file 7: Table S6. The SNPs within miRNA-target binding sites. (XLSX 15 kb)

Additional file 8: Table S7. The SNPs within miRNA-target binding sites between line 9930 and hardwickii. (XLSX 12 kb)

Additional file 9: Figure S2. Real -time PCR detection for the expression of 9930-unique and hardwickii-unique target. (PDF 13136 kb)

Additional file 10: Figure S3. Phylogenetic values of miRNAs-related loci. (PDF 204 kb)

Additional file 11: Table S8. The primers for amplifying precursor sequences of miRNA genes. (XLSX 18 kb)

Additional file 12: Table S9. The primers for 5’RACE. (XLSX 11 kb)

Additional file 13: Table S10. The primers for real-time PCR. (XLSX 10 kb)

## Abbreviations

miRNAs: microRNAs
SNPs: single nucleotide polymorphisms
NAM: No apical meristem
RISCs: RNA-induced silencing complexes
