# Supplementary figures and images for: Genome-wide analysis of microRNA targeting impacted by SNPs in cucumber genome
Source: BMC Genomics. 2017 Apr 4;18:275. doi: 10.1186/s12864-017-3665-y (PMC5379521; doi:10.1186/s12864-017-3665-y)

Color Key

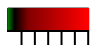

2 8

Value

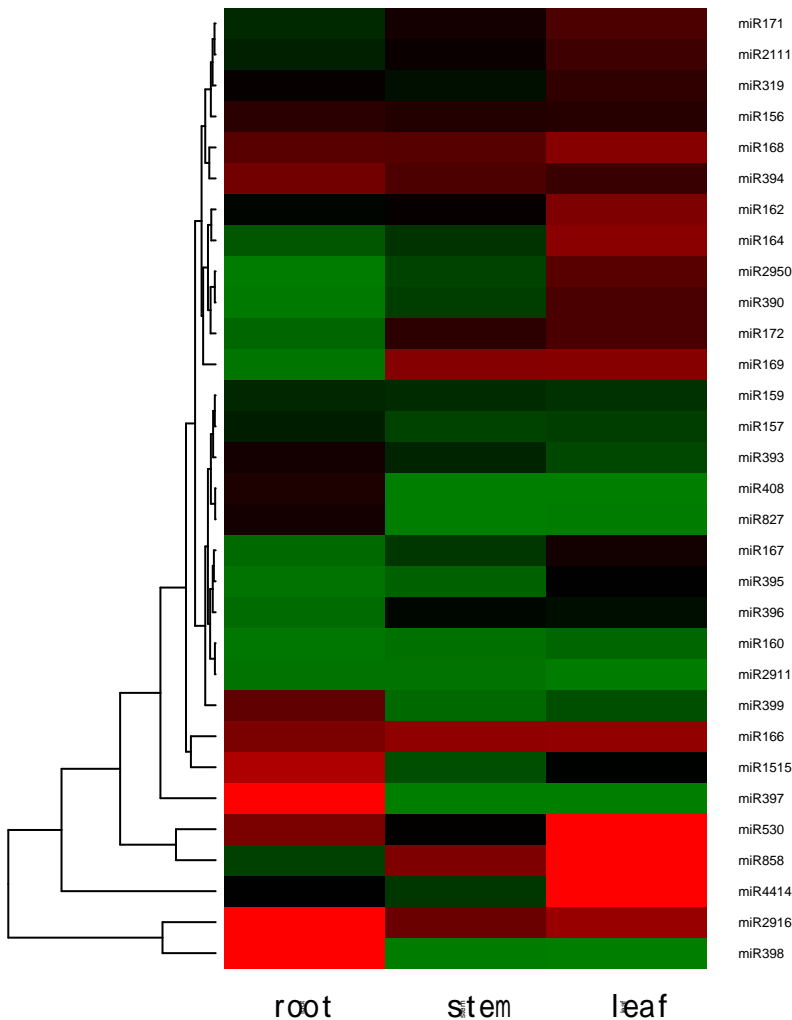

Supplement: Supplementary file 2 — The heat map of miRNAs expression. (PDF 9 kb) [file 12864_2017_3665_MOESM2_ESM.pdf]

SNP in miR166 mature sequence

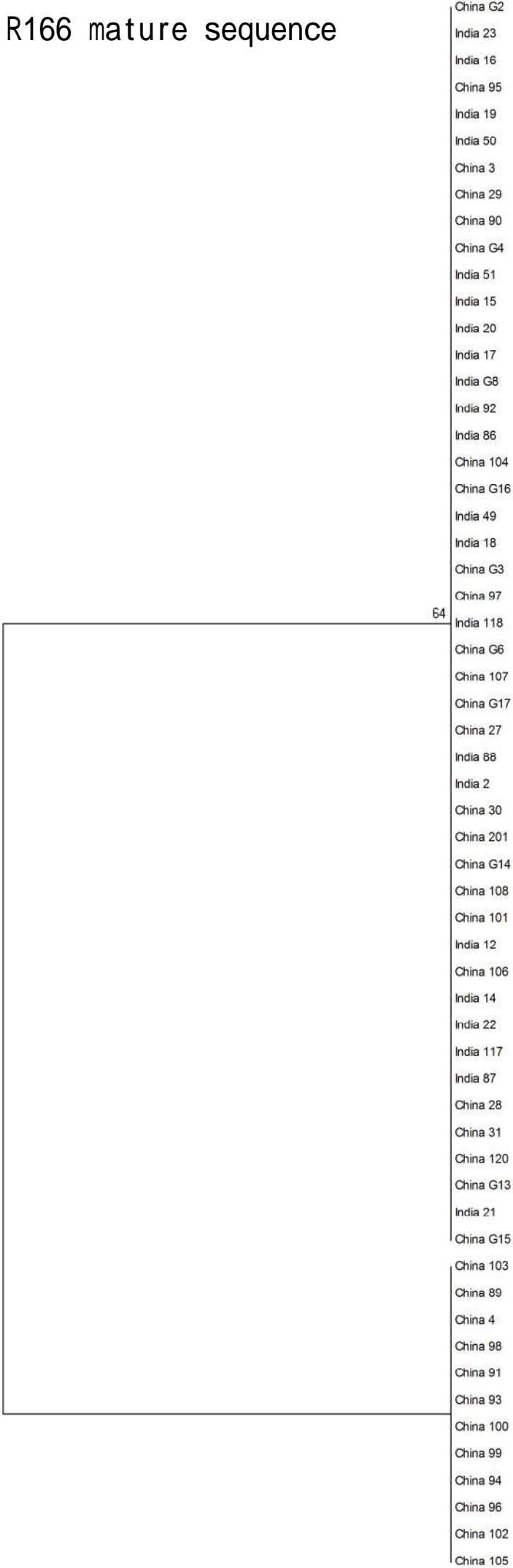

# SNP in miRNA162-target site

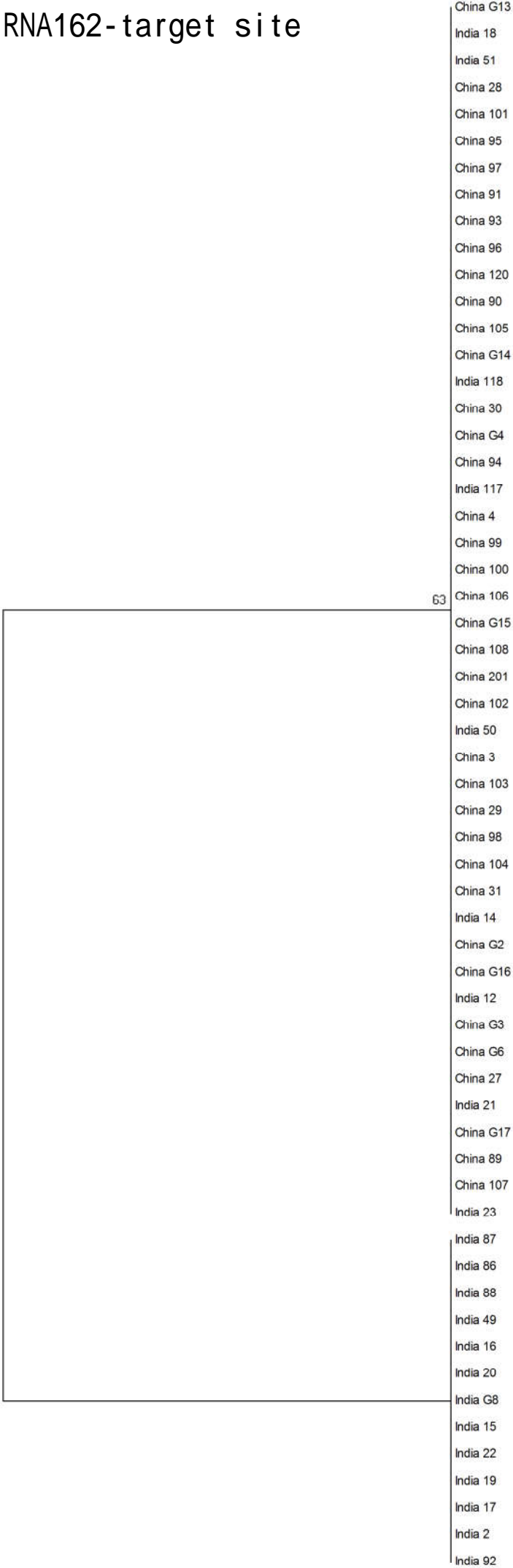

Supplement: Supplementary file 10 — Phylogenetic values of miRNAs-related loci. (PDF 204 kb) [file 12864_2017_3665_MOESM10_ESM.pdf]
