# Supplementary material for: Genome-wide analysis of microRNA targeting impacted by SNPs in cucumber genome
Source: BMC Genomics. 2017 Apr 4;18:275. doi: 10.1186/s12864-017-3665-y (PMC5379521; doi:10.1186/s12864-017-3665-y)

9930-specific targets for conserved and known miRNA

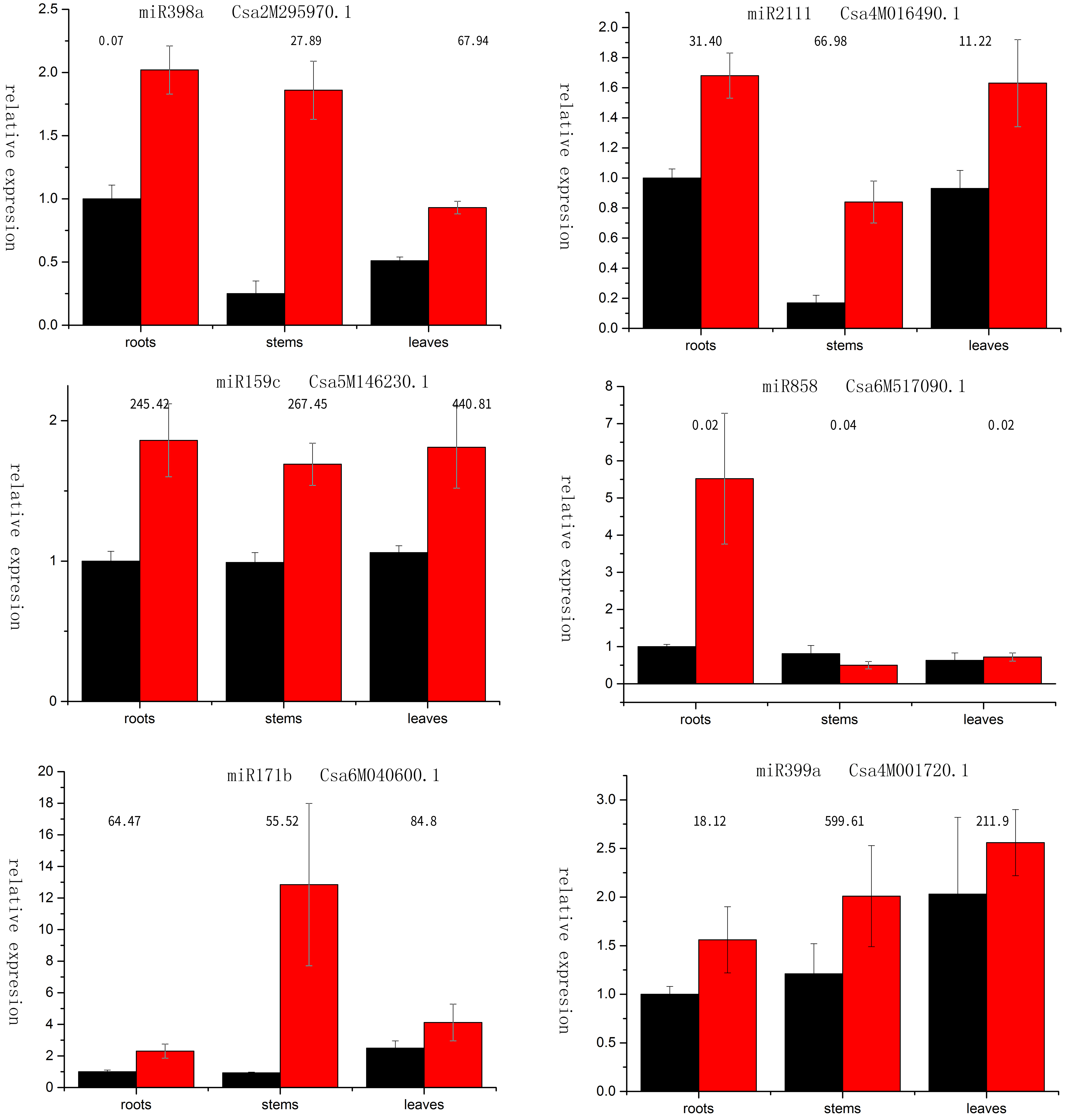

9930-specific targets for cucumber-specific miRNA

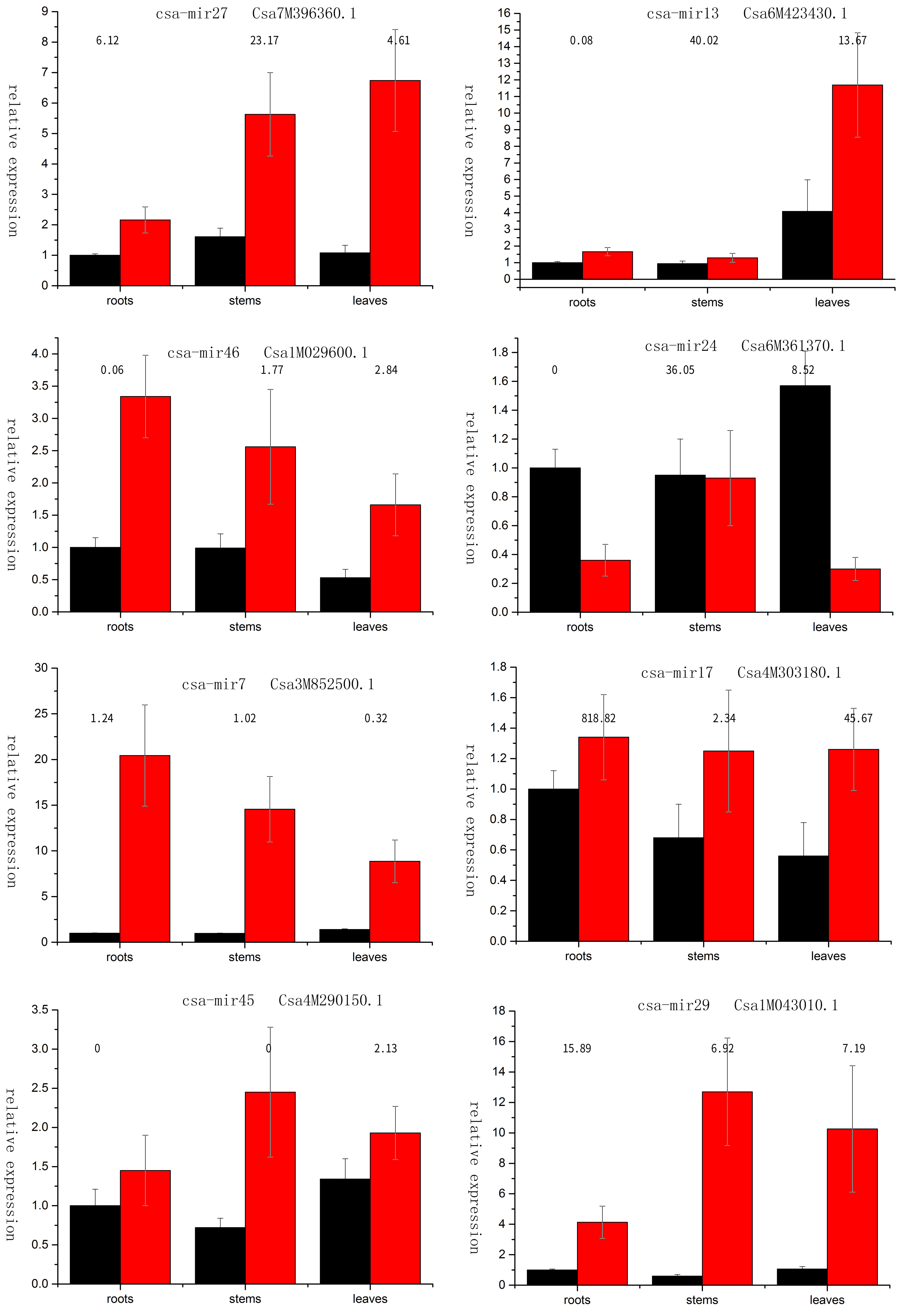

hardwickii-specific targets for conserved and known miRNA

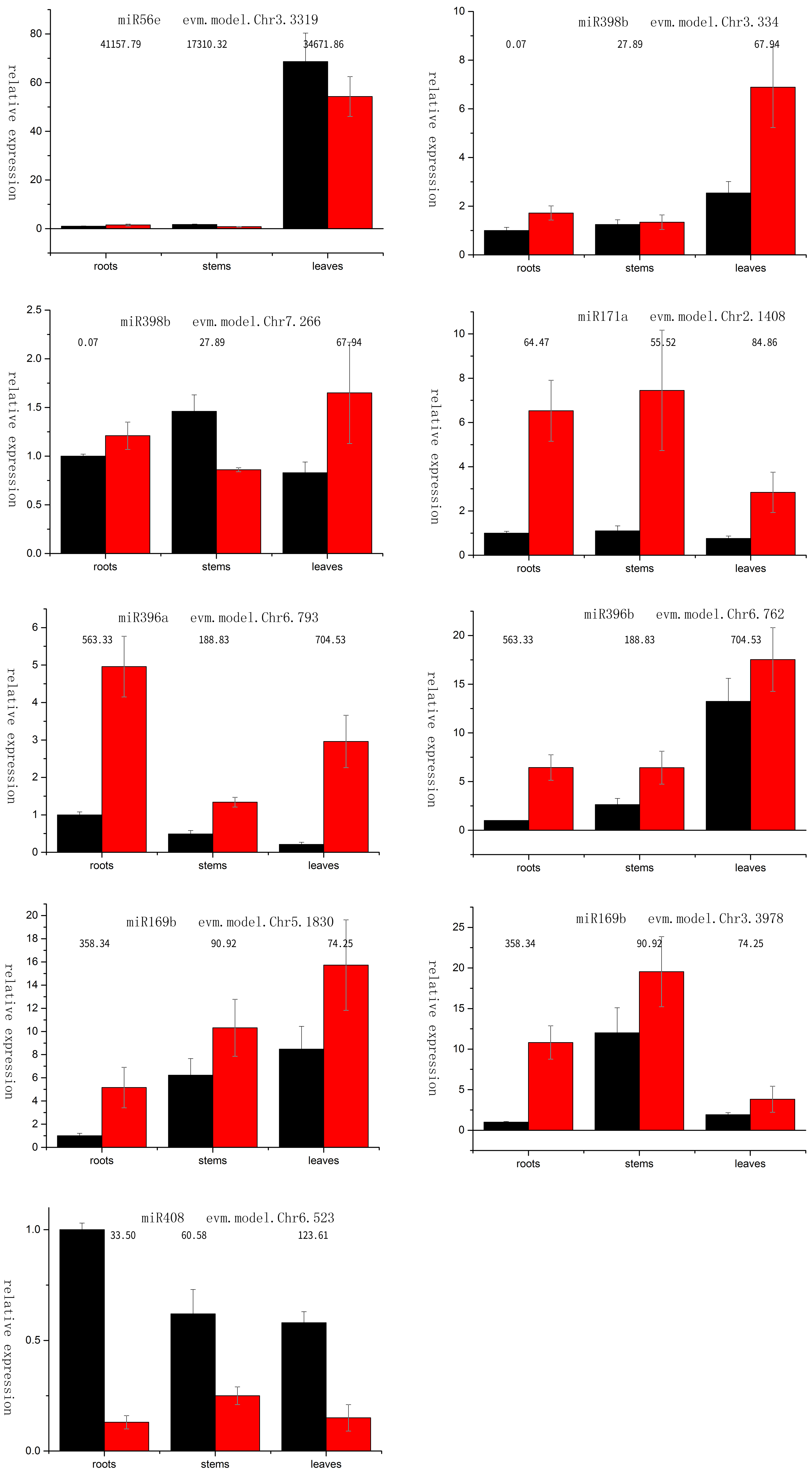

hardwikki-specific targets for cucumber-specific miRNA

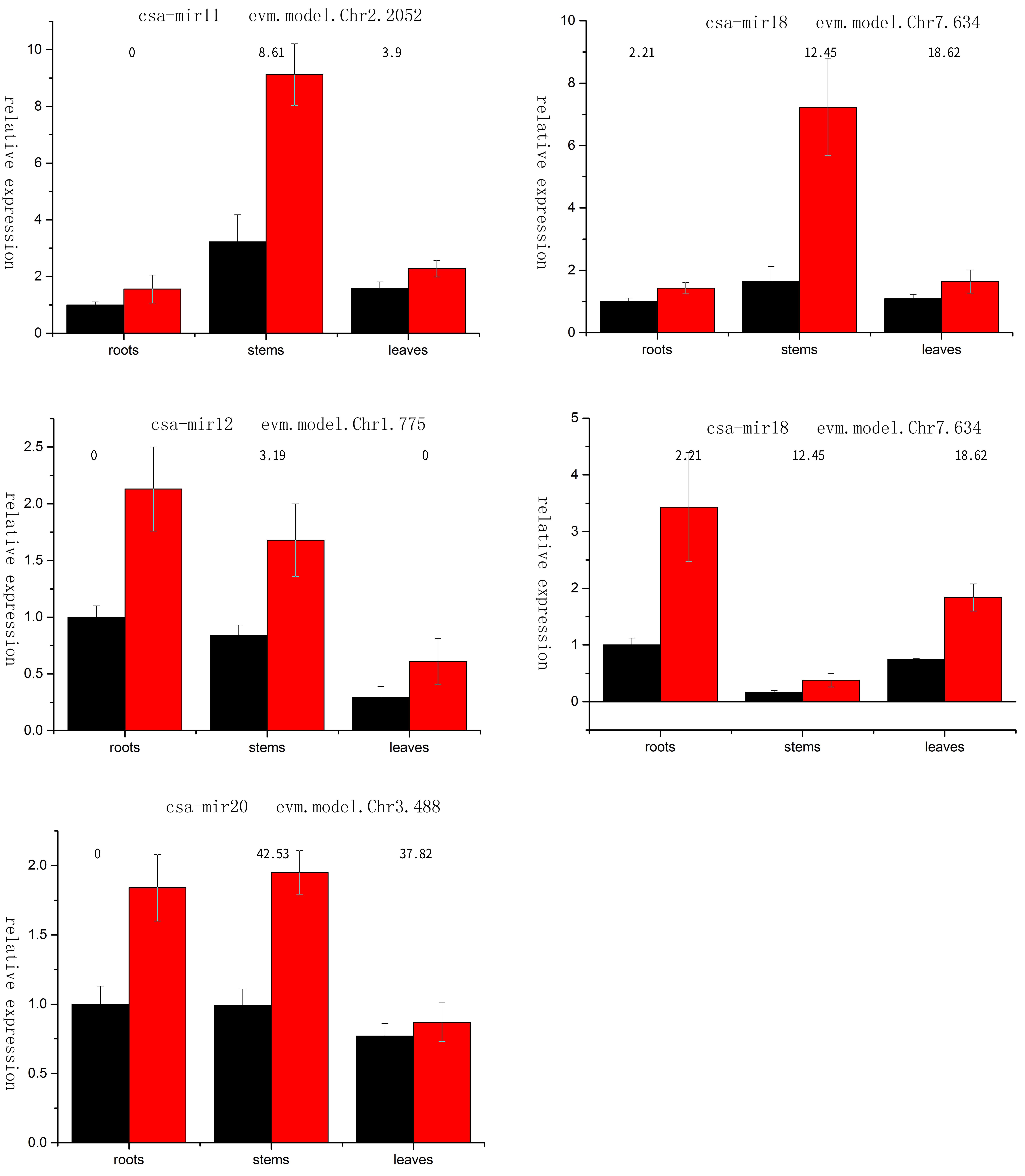

Supplement: Supplementary file 9 — Real -time PCR detection for the expression of 9930-unique and hardwickii-unique target. (PDF 13136 kb) [file 12864_2017_3665_MOESM9_ESM.pdf]
